# Supplementary material for: Subtype-specific characterization of breast cancer invasion using a microfluidic tumor platform
Source: PLoS One. 2020 Jun 16;15(6):e0234012. doi: 10.1371/journal.pone.0234012 (PMC7297326; doi:10.1371/journal.pone.0234012)

**Supplementary Materials**

**Supp. 1** A video clip is provided showing local invasion of SUM-159PT into the collagen matrix.

**Supp. 2** **Invasion characteristics of MDA-MB-231 in IDC-on-chip.** Confocal micrograph of MDA-MB-231 at day 3, cells show membrane protrusions from individual cells as noted with white arrows. Nuclei (blue) and plasma membrane (magenta).


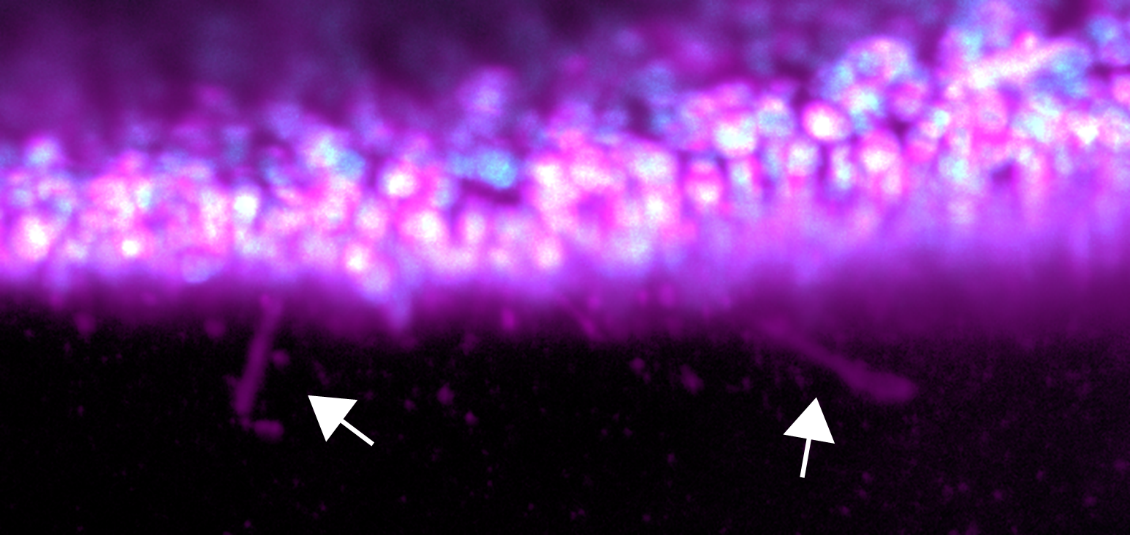

Supplement: S1 File — Confocal micrograph of MDA-MB-231 at day 3, cells show membrane protrusions from individual cells as noted with white arrows. Nuclei (blue) and plasma membrane (magenta). (DOCX) [file pone.0234012.s002.docx]
