# Supplementary material for: Subtype-specific characterization of breast cancer invasion using a microfluidic tumor platform
Source: PLoS One. 2020 Jun 16;15(6):e0234012. doi: 10.1371/journal.pone.0234012 (PMC7297326; doi:10.1371/journal.pone.0234012)
Supplement: Attachment — Submitted filename: Response to Reviewers.pdf [file pone.0234012.s003.pdf]

Reviewer #1's Comments

Comment:

In this paper the authors report a 3D in vitro model of invasive ductal carcinoma using a microfluidic platform. In particular they demonstrate that invasion characteristics of three different human breast cancer cell lines is subtype-specific. The experiments are well performed and the results appear to support the proposed approach. It would be great if the authors could add some experiments with various oxygen concentrations and discussion on effects of hypoxia on invasion characteristics

Response:

We appreciate the reviewer's thoughtful suggestion. We agree that the effect of hypoxia on invasion characteristics can be investigated by using our IDC platform. However, the objective of this study is to compare the invasion characteristics of breast cancer subtypes. We add a discussion about potential use of the developed platform on hypoxia study. (Muñoz-Nájar, Neurath et al. 2006, Chen, Imanaka et al. 2010, Semenza 2012)

Revision:

In page 14:

Throughout the invasion process, distinct biophysical and biochemical features in the tumor microenvironment (TME) such as hypoxia, the existence of fibroblast, various growth factors, and cytokines play critical roles in regulating the cell response. Specifically, the hypoxia has been reported to enhance cancer cell invasion through hypoxia-induced factor (HIF) activities, which regulate the transcription factors such as Snail, Twist, and matrix metalloproteinase. [50-52] Hypoxia induced Notch signaling was reported to mediate epithelial-mesenchymal transition in breast cancer through enhanced expression of Slug and Snail with e-cadherin suppression. [50] With a benefit of the IDC-on-chip platform to recapitulate the physiological architecture of cancer invasion, the platform can be further improved by applying the complex stroma conditions.

Reviewer #2's Comments

This manuscript presents quantitative comparison of invasion process in 3D ductal carcinoma in situ (DCIS) cultures in engineered microfluidic platforms. The authors compared 2D and 3D culture of breast cancer cell lines of MDA-MB-231, SUM-159PT and MCF-7 and proved that their platform could have the invasion prediction potential of breast cancer subtypes. If the platform would realize the closer in-vivo-like microenvironments with co-culture and mechanical stress, then the platform could be a useful tool to characterize and predict invasive potential of breast cancer subtypes or patient-derived cells. The manuscript would seem of considerable interest to those working in cancer cell research in engineered microfluidic platforms. After polished based on the following critiques, this manuscript may be able to be published in PLOS ONE. I would recommend that this paper needs minor revision to be published in PLOS ONE. I recommend the current manuscript should revise to include answers for the questions below:

Comment:

There are several human breast cancer cell lines. Why did authors choose MDA-MB-231, SUM-159PT and MCF-7? In particular, MCF-7 instead of MCF 10? Were the cell lines study in 3D cultures in previous studies? Do the results show the similar to results in the previous work or to in vivo behaviors?

Response:

We selected the three cell lines based on our primary objective to study invasion potential of breast cancer subtypes from mammary duct configuration. MCF-7, MDA-MB-231, and SUM-159PT are subtypes of breast caner cell lines, specifically invasive ductal carcinoma (IDC) which is proceeded after the ductal carcinoma in situ (DCIS). DCIS model using MCF-10 has been reported in literatures. (Bischel, Beebe et al. 2015, Choi, Hyun et al. 2015) Since the developed model intends to mimic IDC, instead of MCF-10, MCF-7 was used as the Luminal A subtype IDC expressing estrogen receptor (ER) and progesterone receptor (PR). The results showed that the MCF-7 barely invaded in the IDC-on chip showing epithelial characteristics similar to MCF-10. Clarification of this point was added in the revision.

Revision:

In page 4:

Since the developed model intends to mimic IDC which is a later stage of the disease after DCIS, the three subtypes of breast cancer cell lines were selected accordingly.

Comment:

The authors evaluated invasion characteristics based on local invasion score. Please describe how to estimate local invasion score and explain why the score is valid to evaluate invasion characteristics.

Response:
Local invasion of cancer cells is a multi-step process including EMT, localized matrix degradation, protrusion, and migration (Friedl and Alexander 2011). Two endpoints we used are relevant to protrusion and migration respectively. The local invasion score was defined as a frequency of sprouting sites per unit length. We measured it by counting the number of sprouting extensions from the lumen boundary. The local invasion score can evaluate invasion characteristics because local invasion frequency illustrates the cell invasion capacity to protrude the adjacent collagen matrix. We clarify it in the manuscript.

Revision:

In page 9:

The sprouts were quantitatively represented as local invasion score and local invasion length, which were defined as a frequency of the sprouting sites and a mean length of the sprouts. The quantitative evaluation of the sprouts with the frequency and length illustrates the invasion capacity of each cell line meticulously.

Comment:

A 3D in vitro microvessel model (Matsunaga et al.) was used in this manuscript. Based on the reviewer's investigation, applying a 3D in vitro microvessel model to breast cancer research is one of the features of this manuscript (Sung et al. Integr Biol 2011; Truong et al. Sci Rep 2016; Choi et al. Lab Chip 2015). If the authors emphasize the benefits of this model to study DCIS, the manuscript would be stronger than the current format.

Response:

We agree the reviewer's suggestion. We revised the manuscript accordingly.

Revision:

In page 4:

Regarding DCIS, several microfluidic models have been proposed to study the effects of fibroblast on the invasion of cancer and non-cancerous epithelial cells of breast tissues. [28-30] The platform in [29] has microchannels where fibroblasts are cultured in collagen to induce chemical cues on

the epithelium of the duct. In addition to the DCIS, an in vitro microfluidic tumor model for the later stage IDC has developed to investigate invasion characteristics of the breast cancer cells. Despite the advances in developing in vitro tumor models, a lack of a reliable model to explore the subtype-specific features of the breast cancer invasion is available.

Comment:

The breast cell invasion responds to ECM stiffness. If the authors describe collagen matrix with the point of view of stiffness, it will help readers to understand microenvironment conditions that induce invasion.

Response:

We agree the reviewer's suggestion. We revised the manuscript accordingly.

Revision:

In page 9:

Since the breast cancer cells invade through the ECM in response to its stiffness, cell sprouts indicating invasion in the IDC-on-chip headed outward of the duct. The stiffness of the collagen matrix at 6 mg/mL has been measured at 0.75 ± 0.12 kPa. [38]
